# Production of Bioadsorbents via Low-Temperature Pyrolysis of Exhausted Olive Pomace for the Removal of Methylene Blue from Aqueous Media

**DOI:** 10.3390/molecules30153254

**Published:** 2025-08-03

**Authors:** Safae Chafi, Manuel Cuevas-Aranda, Mª Lourdes Martínez-Cartas, Sebastián Sánchez

**Affiliations:** 1 Department of Chemical, Environmental and Materials Engineering, Science & Technology Campus of Linares, University of Jaén, Avda. de la Universidad s/n, 23700 Linares, Spain; lcartas@ujaen.es; 2 Olive Grove and Olive Oils Research Institute, 23071 Jaén, Spain; ssanchez@ujaen.es

**Keywords:** exhausted olive pomace, biochar, adsorption, kinetics, isotherms, methylene blue, water treatment

## Abstract

In this work, biochars were produced by pyrolysis of exhausted olive pomace and evaluated as low-cost adsorbents for the removal of methylene blue (MB) from aqueous solutions. The biochar obtained at 400 °C for 1 h, which exhibited the best adsorption performance, was characterized by FTIR, N_2_ adsorption–desorption isotherms, SEM-EDX, and proximate analysis, revealing a mesoporous structure with a relatively low specific surface area but enriched in surface functional groups, likely due to the partial degradation of lignocellulosic components. Adsorption experiments were conducted to optimize operational parameters such as solid particle size (2–3 mm), agitation speed (75 rpm), and bioadsorbent dosage (1 g per 0.05 L of MB solution), which allowed for dye removal efficiencies close to 100%. Kinetic studies showed that MB adsorption followed a pseudo-second-order model, while equilibrium data at 30 °C were best described by the Langmuir isotherm (*R*^2^ = 0.999; *SE* = 4.25%), suggesting monolayer coverage and strong adsorbate–adsorbent affinity. Desorption trials using water, ethanol, and their mixtures resulted in low MB recovery, whereas the addition of 10% acetic acid significantly improved desorption performance. Under optimal conditions, up to 52% of the retained dye was recovered.

## 1. Introduction

The continuous expansion of both global population and industrial activities has significantly contributed to the release of hazardous substances into the environment, particularly affecting water, air, and soil quality. Among the wide range of pollutants, synthetic organic dyes have attracted considerable attention due to their chemical stability, toxicity, and resistance to biodegradation. These compounds are widely employed in numerous industrial sectors, including textile manufacturing, printing, plastics, leather processing, cosmetics, pharmaceuticals, and food production [1]. It is currently estimated that over 100,000 commercial dyes are available on the global market, with an annual consumption exceeding 700,000 tons. During textile dyeing and finishing processes, approximately 15% of the dyes used are lost to wastewater streams, ultimately entering aquatic ecosystems [2]. According to reports from the Organization for Economic Cooperation and Development, the textile sector ranks among the most water-intensive industries worldwide [3]. Furthermore, projections indicate that wastewater pollution from textile and related industries may double by the year 2050, positioning this sector as the second-largest contributor to global water pollution, surpassed only by agriculture [4].

Within this context, methylene blue (MB), a cationic thiazine dye, stands out due to its widespread use and hazardous environmental profile. MB exhibits high water solubility and structural stability, which facilitate its accumulation in water bodies. Once present in aquatic environments, MB can hinder light penetration and reduce dissolved oxygen levels, thereby disrupting aquatic ecosystems. From a human health perspective, prolonged exposure to methylene blue, particularly at elevated concentrations, has been associated with adverse effects, including genitourinary and gastrointestinal disorders, as well as disturbances to the central nervous and respiratory systems [5]. Consequently, there is a growing need to explore innovative, cost-effective, and environmentally sustainable methods for the removal of persistent organic pollutants from water and wastewater systems, with particular emphasis on safeguarding clean water resources. Conventional treatment of industrial wastewater generally involves biological, chemical, and physical processes, including enzymatic degradation, microbial biomass treatment, advanced oxidation processes (AOPs), Fenton reactions, electrochemical oxidation, membrane filtration, coagulation–flocculation, ion exchange, chemical precipitation, and reverse osmosis. However, these techniques often present significant drawbacks, such as high operational costs, the requirement for specialized equipment, excessive use of chemical reagents, generation of secondary pollutants or sludge, and prolonged treatment times [6]. In contrast, adsorption has emerged as a promising alternative due to its operational simplicity, low cost, minimal environmental impact, and high efficiency in removing colored contaminants from aqueous solutions [7]. In recent years, particular attention has been directed towards the use of biomass-derived adsorbents (often referred to as bioadsorbents) due to their abundance, renewability, biodegradability, low cost, and high adsorption potential [6]. Various lignocellulosic materials have been successfully applied for the adsorption of methylene blue from contaminated water, including rice straw [8], avocado seed [9], black and green olive stones [10], olive stone [11], Maclura pomifera and wild carob [12], banana peels [13], coconut shells [14], and Moringa oleifera [15].

Among the biomass residues abundantly generated by Spanish agriculture, olive oil by-products are particularly prominent due to their economic significance, local-level production, and renewability. Spain is the world’s leading producer of olive oil, with more than a million tons per year [16], encompassing the largest olive cultivation area, totaling 2,507,684 hectares with over 180 million trees. The province of Jaén (2.67% of the national territory), situated in Andalusia (southern Spain), is the most productive and accounts for almost a quarter of Spain’s olive grove area and about 42% of the cultivated land in Andalusia [17]. During olive oil production, considerable quantities of by-products are generated, including olive stones (pits) and olive pomace. The latter undergoes further processing to extract additional oil with hexane, resulting in exhausted olive pomace (EOP), which is subsequently utilized as biomass for energy production [18]. The average annual generation of exhausted olive pomace in Andalusia is estimated to range between 1.2 and 1.5 million tons [19]. For this reason, a valuable opportunity exists to enhance the economic and environmental value of this low-cost by-product through adsorption techniques, enabling the removal of contaminants from environmental media. In the literature, references can be found regarding the use of olive oil pomace as an adsorbent medium for methylene blue [20]. However, in that study, the raw material was subjected to oil extraction using ethyl acetate, a solvent not used in current industrial practice, which would limit the potential food-grade use of the extracted oil.

The thermochemical treatment of biomass, through methods like gasification, pyrolysis and torrefaction produces biochar as a solid residue. A significant benefit of biochar is its biodegradability and its reduced ecotoxicity compared to nanoparticle adsorbents [21]. Activated carbon is a high-performance adsorbent with excellent capabilities for removing various dyes and metals, but its high cost is a significant drawback. In contrast, plant-derived biochar (especially those generated at moderate temperatures) could offer similar adsorption capabilities to commercial activated carbon, along with the advantages of ample availability, affordability, and environmental friendliness [22].

Numerous investigations have demonstrated that biochar is highly effective in adsorbing micropollutants from water. Abdoul-Latif et al. reported that olive pomace biochar, produced at 450 °C, effectively adsorbed total phenolic compounds from olive mill wastewater, with a maximum capacity of 66.67 mg g^−1^ compared to 21.27 mg g^−1^ for raw olive pomace [23]. Kan et al. prepared biochars from coffee shells through pyrolysis, with the water-washed sample produced at 700 °C demonstrating a notably high adsorption capacity of 193.5 mg g^−1^ for the removal of Rhodamine B from wastewater [24]. Another recent study investigates the simultaneous removal of As(III) and oxytetracycline (OTC) using a novel bimetallic zinc–iron-modified biochar, which demonstrated strong adsorption capacities of 34.7 mg g^−1^ for As(III) and 172.4 mg g^−1^ for OTC [25]. On the other hand, garlic peel was evaluated as an effective biochar for the removal of methylene blue, with the sample pyrolyzed at 150 °C exhibiting the highest adsorption capacity of 167.74 mg g^−1^ [26].

In the present study, the use of a low-cost bioadsorbent, specifically biochar obtained by low-temperature pyrolysis (200–500 °C) of EOP, is analyzed for the first time for the adsorption of methylene blue from aqueous solutions. First, the effect of pyrolysis conditions (time and temperature) on the adsorptive capacity of the biochar is evaluated. Once the most suitable solid for dye removal is identified, the influence of various factors, such as particle size, agitation speed, solid dosage, and initial solute concentration, on the methylene blue adsorption capacity is investigated. In addition, preliminary desorption tests of methylene blue adsorbed onto biochar are conducted.

## 2. Results and Discussion

### 2.1. Production of Bioadsorbents by Low-Temperature Pyrolysis

**Figure 1:**
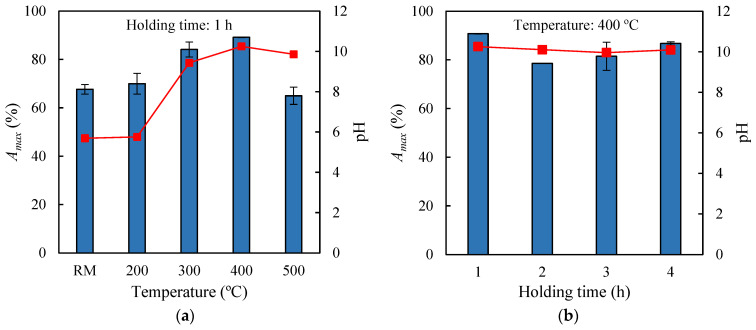
pH of the adsorption medium (dots) and maximum adsorption percentages of methylene blue (bars) onto the raw material (RM) and the corresponding biochars obtained at different pyrolysis temperatures (**a**) and holding times (**b**).

To determine the effect of low-temperature pyrolysis treatment on the adsorption capacity of exhausted olive pomace (EOP), four thermal treatments of the raw material were first carried out using maximum temperatures of 200 °C, 300 °C, 400 °C, and 500 °C, each maintained for 1 h. The solid recovery yields were 96.5%, 53.1%, 40.6%, and 36.3%, respectively, indicating, on one hand, that no significant changes in the biomass structure occurred at 200 °C (polymer degradation in biomass typically begins at temperatures above 200 °C [27]), and on the other hand, that increasing the temperature led to a continuous decrease in bioadsorbent yield, likely due to greater generation of volatile materials. The solid recoveries obtained at 400 °C and 500 °C were similar but slightly higher than those reported in the literature for the pyrolysis of exhausted olive waste at the same temperatures, at 31.7% and 27.3%, respectively [28]. The four biochars, along with the raw material, were used as adsorbent materials in assays conducted at 30 °C with an initial methylene blue (MB) concentration of 10 mg L^−1^, adsorbent load of 0.5 g per 0.05 L MB solution, and agitation speed of 150 rpm. Adsorption was carried out for 24 h to ensure that equilibrium conditions were reached and the final pH of the adsorption medium was measured. The results are shown in Figure 1a, where it can be observed that the untreated raw material (EOP) retained 67.7% of the dye, while the biochars obtained at 200 °C, 300 °C, 400 °C, and 500 °C achieved maximum adsorption percentages (*A_max_*) of 70.0%, 84.1%, 89.1%, and 65.0%, respectively. Therefore, the highest adsorption capacity was obtained with the biochar generated at 400 °C, representing a 32% increase compared to the adsorption achieved with the raw material. Figure 1a also highlights, on the one hand, the existence of significant differences in the pH of the adsorption medium (ranging from 5.7 to 10.3) depending on the type of biochar used, and on the other hand, that the highest adsorption capacity was achieved at higher pH values. These variations in pH can be attributed to the ability of certain types of biochar to modify the pH of an aqueous medium, owing to the presence of surface functional groups and mineral compounds that act as alkalinizing or acidifying agents [29]. Furthermore, to determine the effect of pyrolysis time on the adsorptive properties of the biochars, experiments were conducted where EOP was thermally treated at 400 °C with holding times of 1, 2, 3, and 4 h. These treatments resulted in solid recoveries of 42.6%, 40.3%, 40.9%, and 38.9%, respectively. When used as bioadsorbents under the same operational conditions previously mentioned, the biochars achieved *A_max_* values shown in Figure 1b, ranging from 78.6% to 90.8%, while the pH values of the liquid media ranged between 10.0 and 10.3. It is thus inferred that, at 400 °C, increasing the pyrolysis time from 1 to 4 h did not lead to improvements in the methylene blue retention capacity of the biochars. Consequently, the biochar generated from EOP by pyrolysis at 400 °C for 1 h was selected as the best bioadsorbent. This biochar will hereafter be referred to as EOPB.

### 2.2. Characterization of the Raw Material and Biochars

To better understand the effect of pyrolysis on the raw material, proximate and ultimate analyses were carried out to evaluate the compositional changes in exhausted olive pomace (EOP) and its derived biochars produced at 400 °C and 500 °C with a holding time of 1 h (Table 1). The proximate analysis revealed that the raw material (EOP) exhibited a relatively high ash content (9.5%), which increased significantly with pyrolysis temperature, reaching 25.6% in the biochar generated at 500 °C. This progressive ash enrichment was accompanied by a consistent decrease in volatile matter and a corresponding increase in fixed carbon, reflecting enhanced thermal decomposition and aromatization. These trends are in line with previous reports on the pyrolysis of EOP at similar conditions [30]. Regarding the ultimate analysis, the increase in pyrolysis temperature from 400 °C to 500 °C resulted in an increase in carbon content in the biochars, whereas the contents of hydrogen and oxygen decreased (Table 1), indicating a higher degree of carbonization. However, it is important to highlight that the actual elemental composition is also influenced by the accumulation of mineral matter during pyrolysis. As previously noted, the ash content in the biochars was substantially higher than in the raw biomass. To gain further insight into the elemental distribution, EDX-SEM analysis was performed on the three samples, and the corresponding micrographs and spectra are provided in Figures S1–S3 (see Supplementary Material). The transformation of EOP into biochar led to a continuous decrease in carbon content (from 70.9% in EOP to 63.5% and 62.8% in the biochars obtained at 400 °C and 500 °C, respectively), accompanied by a similar decline in oxygen content (from 24.6% to 23.9% and 18.3%, respectively). The content of inorganic compounds increased markedly with pyrolysis temperature, which may account for the rise in the pH of the aqueous medium observed when using the biochars produced at 400 °C and 500 °C for 1 h (Figure 1a). The EDX analysis of the 400 °C × 1 h biochar revealed the following elemental composition: 7.3% K, 1.8% Ca, 1.4% Si, 0.8% Mg, 0.3% P, 0.3% Al, 0.4% Cl, and 0.2% Fe. In contrast, the 500 °C × 1 h biochar contained 10.4% K and 4.1% Ca. These results are in agreement with previous studies on EOP pyrolysis, which identified potassium and calcium as the predominant inorganic elements in its biochar [31]. Interestingly, although the biochar obtained at 500 °C had a higher inorganic content than that produced at 400 °C, both materials led to similar pH values (around 10) in the aqueous phase (Figure 1a), possibly due to the establishment of an equilibrium in the ion transfer between the solid and liquid phases.

**Figure 2:**
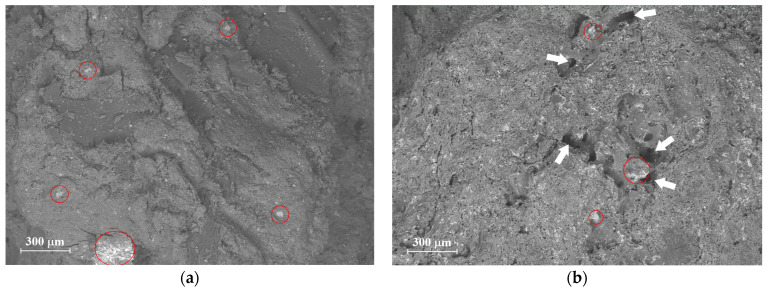
SEM images of exhausted olive pomace (**a**) and the biochar obtained from it at 400 °C for 1 h (**b**). Some mineral matter particles are indicated with circles, while the arrows point to pores formed in the biochar.

The release of volatile matter during biomass pyrolysis typically results in an increase in specific surface area due to the development of new pores. To evaluate this phenomenon, the specific surface area (SSA) and Average Pore Diameter (APD) were determined via nitrogen adsorption isotherms. The EOP sample exhibited SSA and APD values of 0.188 m^2^ g^−1^ and 33.675 Å, respectively, while the EOPB sample showed values of 0.443 m^2^ g^−1^ and 55.04 Å. These results indicate a predominance of mesopores in both materials, with the thermal treatment inducing a moderate increase in both SSA and APD. The impact of pyrolysis on surface morphology was further assessed by comparing SEM micrographs of the raw biomass (Figure 2a) and the pyrolyzed solid at 400 °C for 1 h (Figure 2b). The pyrolyzed material exhibited a more irregular surface, attributed to the formation of numerous pores, some of which (indicated by an arrow in Figure 2b) reached diameters of approximately 100 microns. In Figure 2, some surface areas of both EOP and EOPB with a high presence of mineral matter are also highlighted using circles.

**Figure 3:**
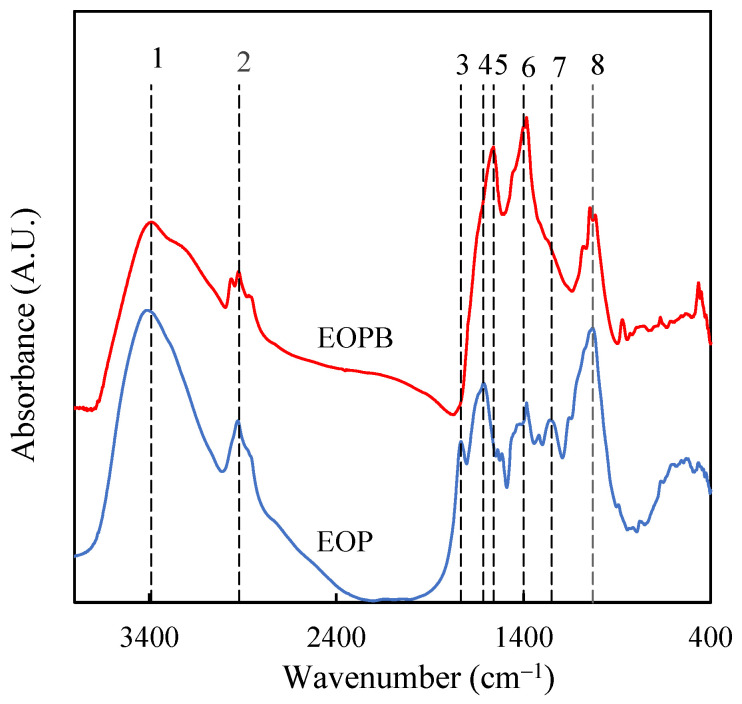
FTIR spectra of exhausted olive pomace (EOP, blue line) and its derived biochar obtained at 400 °C for 1 h (EOPB, red line).

To complete the characterization of EOP and EOPB, the functional groups present on their surfaces were identified by FTIR analysis (Figure 3). For the raw material, the FTIR spectrum closely resembles that reported by other authors for two-phase olive mill solid waste [32].The main absorption bands appeared at 3390 cm^−1^ (position 1 in Figure 3), commonly associated with O–H stretching vibrations from water and polysaccharides in the sample; 2920 cm^−1^ (position 2), usually attributed to C–H stretching vibrations from methyl and methylene groups; 1735 cm^−1^ (position 3), which could correspond to C=O stretching vibrations in ketone, carbonyl, and carboxyl groups [33]; 1615 cm^−1^ (position 4), likely related to aromatic C=C stretching in lignin [34]; 1400 cm^−1^ (position 6), a band that has been associated with aromatic skeletal vibrations in lignins [35]; 1250 cm^−1^ (position 7), attributed to C–O–C bonds in acetyl groups [36,37]; and 1030 cm^−1^ (position 8), often ascribed to C–O bonds in alcohols. A comparison of the spectra of the raw material and EOPB reveals that the thermal treatment led to a decrease in the intensity of the band at position 1, possibly due to the lower holocellulose content in the biochar, which also reduces the moisture content by increasing its hydrophobic character [38]. The disappearance of the spectral peaks at positions 3 and 7 in the biochar, along with the marked decrease in the band at position 8, may indicate significant transformation of both hemicellulose and cellulose. In this regard, it has been reported that during the pyrolysis of two-phase olive mill solid waste, hemicellulose and cellulose decomposition occurs at temperatures around 250–300 °C and 300–400 °C, respectively, whereas higher temperatures (400–600 °C) are required to transform most of the lignin [39]. Therefore, pyrolysis of EOP at 400 °C for 1 h resulted in a material in which the FTIR signals associated with oxygenated functional groups were reduced, while those related to aromaticity were intensified, such as the bands located at positions 6 (1400–1380 cm^−1^) and 5 (1560 cm^−1^), the latter being attributed to C=C stretching in aromatic rings [24,30].

### 2.3. Influence of Particle Size and Agitation Speed

**Figure 4:**
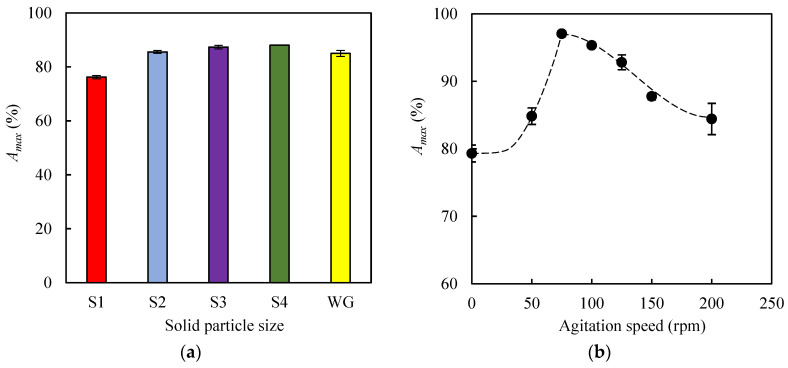
Maximum percentages of methylene blue adsorbed on biochar obtained at 400 °C for 1 h. (**a**) Influence of particle size: S1, 0.4–0.6 mm; S2, 0.6–1.0 mm; S3, 1.0–2.0 mm; S4, 2.0–3.0 mm; WG, without grinding. (**b**) Influence of agitation speed.

In this experimental series, the influence of both particle size and agitation speed on methylene blue (MB) adsorption was studied using, as the adsorbent, the biochar produced by pyrolysis of EOP at 400 °C for 1 h (EOPB solid). In all adsorption experiments within this section, the following operational conditions were kept constant: temperature (30 °C), initial MB concentration (10 mg L^−1^), and adsorbent load (0.5 g per 0.05 L of MB solution). The *A_max_* (%) values were determined after 24 h of adsorption. Particle size is a variable that typically affects mass transfer processes between solid and liquid phases. For this reason, a fraction of pelletized EOPB was ground using a porcelain mortar, and the resulting solids were sieved using commercial sieves (Filtra Vibración). In this way, four particle size ranges were obtained, namely 0.4–0.6 mm, 0.6–1.0 mm, 1.0–2.0 mm, and 2.0–3.0 mm, which were assigned the codes S1, S2, S3, and S4, respectively. Figure 4a shows the *A_max_* values obtained for the experiments carried out with solids S1-S4 and with the pelletized biochar of original size (without grinding, WG), which had an average length of 18.9 ± 1.3 mm and an average diameter of 4.9 ± 0.1 mm. These dimensions are significantly smaller than those reported for the raw material in Section 3.1, which may be attributed to the shrinkage experienced by the solid during the pyrolysis process [40]. The highest *A_max_* value (88.1%) was obtained with solid S4 and was slightly higher than that achieved with the pelletized biochar (85.0%). However, reducing the particle size below 2.0 mm led to a continuous decrease in the adsorption percentage, with the lowest *A_max_* value (76.2%) observed for the smallest particle size (S1). This behavior may be attributed to the fact that, during the biomass grinding and sieving processes, the finer fractions became enriched in inorganic matter, as evidenced by the ash contents of 23.8%, 22.3%, and 21.6% in fractions S1, S2, and S3, respectively. This increased mineral content could potentially impair their adsorption capacity due to a reduction in the availability of active carbonaceous sites. Based on these results, the particle size range between 2 mm and 3 mm was selected as the most suitable for the subsequent adsorption experiments.

On the other hand, to determine the influence of agitation speed on the *A_max_* value, seven agitation speeds were tested (0 rpm, 50 rpm, 75 rpm, 100 rpm, 125 rpm, 150 rpm, and 200 rpm). It is well known that increasing the agitation speed of the flasks leads to an increase in turbulence in the liquid medium and in the Reynolds number (*Re*). This dimensionless number was calculated following the methodology described by Tan et al. [41]. For this purpose, the density and viscosity of the methylene blue solutions at 30 °C were assumed to be practically equal to those of water (1000 kg m^−3^ and 7.97 × 10^−4^ Pa·s, respectively), since low dye concentrations were used. Considering that the maximum internal diameter of the flasks was 6 × 10^−2^ m, Reynolds numbers in the range of 2.26 × 10^5^ to 9.03 × 10^5^ were obtained for agitation speeds between 50 rpm and 200 rpm, which implies a turbulent flow regime in all cases, as *Re* values above 6 × 10^4^ are indicative of turbulence [42]. Regarding the *A_max_* values, Figure 4b shows that the highest adsorption percentage (97.1%) was achieved at an agitation speed of 75 rpm, while at both 0 rpm and 200 rpm the percentages were significantly lower (79.3% and 84.4%, respectively). This type of behavior has been reported in other adsorption studies where weak physical interactions between solute and adsorbent are established, so that increasing system turbulence beyond a critical value (in this case, 75 rpm; *Re* = 3.39 × 10^5^) could affect the stability of the adsorbent–adsorbate bonds and, consequently, the adsorption efficiency. In this regard, Cuevas et al. found that an agitation speed of 80 rpm led to the highest *A_max_* values during the adsorption of furfural onto lignin-rich biomasses [43].

### 2.4. Effect of Adsorbent Load

**Figure 5:**
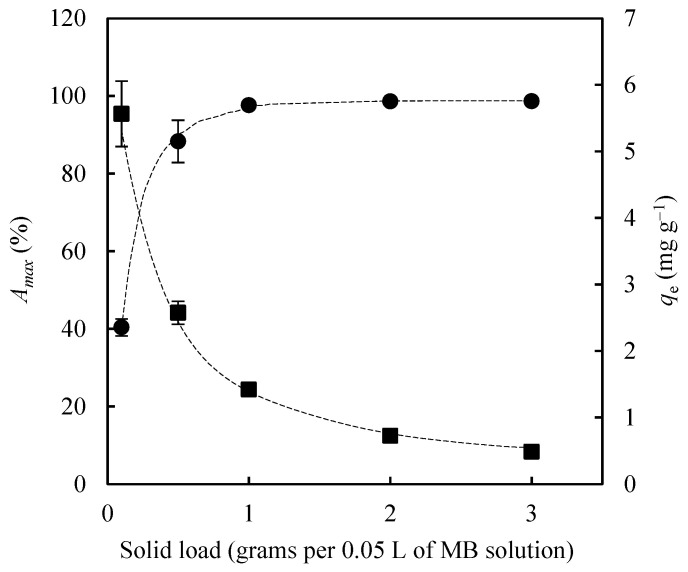
Influence of adsorbent load on the maximum methylene blue adsorption percentage (*A_max_* %, circles) and on the equilibrium adsorption capacity (*q_e_* mg g^−1^, squares).

Adsorbent loading is one of the most influential parameters in adsorption processes, as higher solid dosages increase the availability of active sites for solute binding. Accordingly, a series of adsorption experiments was carried out using EOPB particles with a size range of 2–3 mm. The experimental conditions were maintained constant: adsorption temperature at 30 °C, agitation speed at 75 rpm, initial methylene blue (MB) concentration at 30 mg L^−1^, and bioadsorbent dosages of 0.1, 0.5, 1.0, 2.0, and 3.0 g per 0.05 L of MB solution. Both the maximum adsorption percentage (*A_max_*, %) and the maximum adsorption capacity (*q_e_*, mg g^−1^) were determined after 24 h of contact. As shown in Figure 5, increasing the amount of biochar in the Erlenmeyer flask resulted in a progressive rise in *A_max_* (from 40.4% to 98.7%) and a corresponding decline in *q_e_* (from 5.57 mg g^−1^ to 0.49 mg g^−1^). Furthermore, dosages of 1.0, 2.0, and 3.0 g yielded comparable maximum MB removal efficiencies (97.6%, 98.6%, and 98.7%, respectively). Consequently, 1.0 g of biochar per 0.05 L of MB solution was selected as the optimal dosage for subsequent experimental runs.

### 2.5. Kinetic Study and Adsorption Isotherm

**Figure 6:**
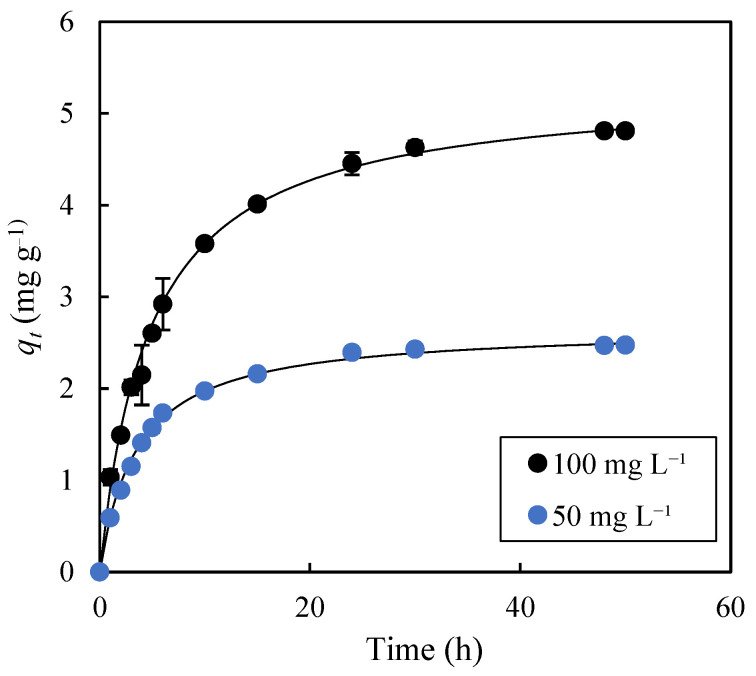
Time evolution of the experimental *q_t_* values for methylene blue adsorption onto biochar produced at 400 °C for 1 h. The solid lines represent the fitting of the experimental data (dots) to the pseudo-second order model. Adsorption conditions: agitation speed, 75 rpm; temperature, 30 °C.

The study of adsorption kinetics is of great practical interest, as it determines, among other aspects, the volume of the equipment where the separation process will be carried out. Figure 6 shows the evolution of the parameter *q_t_* over 50 h of adsorption for initial solute concentrations of 50 mg L^−1^ and 100 mg L^−1^. It can be observed that increasing the initial solute concentration resulted in a longer time to reach equilibrium [44]. Thus, while 24 h were needed to reach equilibrium with *C*_0_ = 50 mg L^−1^, a significantly longer time (48 h) was required with an initial solute concentration of 100 mg L^−1^. The experimental data were fitted to four kinetic models: pseudo-first-order (PFO), pseudo-second-order (PSO), Weber–Morris (WM), and Elovich. Table 2 lists the parameters obtained from these fittings. The PSO model, with *R*^2^ values of 1.000 and *SE* values of 2.3% and 3.4%, provided the best fit compared to the PFO model (*R*^2^: 0.991 and 0.992; *SE*: 35.7% and 36.0%), the Weber–Morris model (*R*^2^ in the range 0.985–0.994; *SE* in the range 1.7–9.9%), and the Elovich model (*R*^2^: 0.982 and 0.965; *SE*: 6.2% and 7.8%). For the WM model, two values of *K_df_* (*K_df_*_1_ y *K_df_*_2_) are reported because two stages with different kinetic behaviors were considered. The first stage extended from the initial time to 6 h and 10 h of adsorption for the experiments with initial MB concentrations of 50 mg L^−1^ and 100 mg L^−1^, respectively. The fact that the *SE* values for the PSO model were below 5%, and also lower than those of the PFO, WM, and Elovich models, confirms the high fitting accuracy of the PSO model to the experimental data [45]. From Equation (5), by calculating the derivative of *q_t_* with respect to time, at *t* = 0, the product *q_e_*^2^*K*_2_ was obtained, allowing for the determination of the initial adsorption rates in the experiments with initial methylene blue concentrations of 50 mg L^−1^ and 100 mg L^−1^. These values were 0.761 mg g^−1^ h^−1^ and 1.0951 mg g^−1^ h^−1^, respectively, and they demonstrate that increasing the initial solute concentration led to higher initial adsorption rates. The same behavior, but with higher initial adsorption rates (1.939 mg g^−1^ h^−1^ for *C*_0_ = 50 mg L^−1^, and 1.0951 mg g^−1^ h^−1^ for *C*_0_ = 100 mg L^−1^), was observed based on the *α* parameter of the Elovich model (Table 2). The initial adsorption rate values reveal a slow kinetics for the adsorption of methylene blue onto exhausted olive pomace biochar, although this kinetic behavior is of the same order of magnitude as that reported by other authors [46] for the adsorption of Zn ions onto apple wood biochar produced at 300 °C (*α* = 5.26 mg g^−1^ h^−1^).

**Figure 7:**
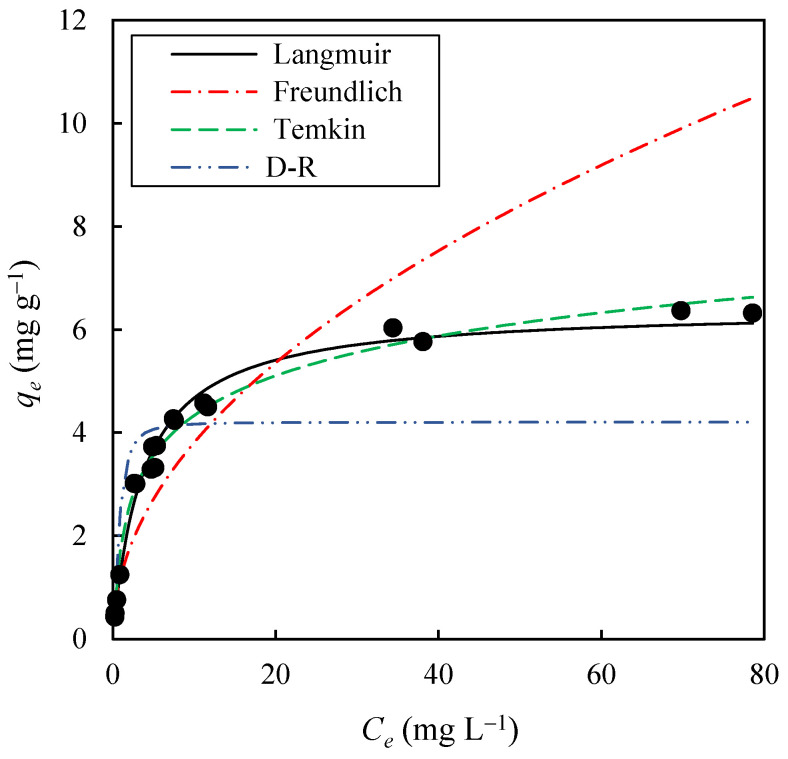
Experimental values (black dots) of *C_e_* vs. *q_e_* for methylene blue adsorption at 30 °C onto biochar obtained at 400 °C for 1 h, and fitting of the experimental data to the Langmuir, Freundlich, Temkin, and Dubinin-Radushkevich (D-–R) isotherm models (lines).

On the other hand, the effect of the initial solute concentration on the equilibrium adsorption of methylene blue (MB) onto EOPB was studied at 30 °C. For this purpose, 19 solutions with initial dye concentrations (*C*_0_) ranging from 10 to 200 mg L^−1^ were prepared, and the adsorption process was carried out until no further changes in MB concentration were observed, indicating that equilibrium had been achieved. In this way, experimental values of *C_e_* vs. *q_e_* were obtained, as shown in Figure 7. The equilibrium concentrations (*C_e_*) ranged from 0.27 mg L^−1^ to 78.58 mg L^−1^, while the equilibrium adsorption capacities (*q_e_*) varied between 0.43 mg g^−1^ and 6.37 mg g^−1^. Additionally, it can be observed that the increase in *C_e_*, from 0.27 mg L^−1^ to 11.67 mg L^−1^, led to a much greater increase in *q_e_* than the one achieved for *C_e_* values above 20 mg L^−1^. Table 3 presents the results obtained from fitting the experimental data to the Langmuir (Equation (8)), Freundlich (Equation (10)), Temkin (Equation (11)), and Dubinin-Radushkevich (Equation (12)) adsorption isotherms. These results indicate that the Langmuir isotherm provides the best fit to the equilibrium data, with *R*^2^ values of 0.999 and a standard error (*SE*) of 4.25%, while for the Temkin, Dubinin-Radushkevich, and Freundlich models, *SE* values of 9.77%, 24.47%, and 30.13%, respectively, were obtained. As shown in Figure 7, the solid black line, corresponding to the Langmuir model, achieves the best fit to the equilibrium data. This suggests that MB adsorption onto the EOPB surface occurs via monolayer formation, which limits the maximum adsorption capacity of the adsorbent to a value equal to *q_m_* (6.414 mg g^−1^, Table 2), and that the adsorbent surface is relatively homogeneous in terms of adsorption energy. Furthermore, the adsorbent–adsorbate equilibrium constant (*K_L_*), which reflects the affinity of the adsorbate for the adsorbent, was found to be 0.269 L mg^−1^ (Table 2). Based on the *K_L_* and *C*_0_ values, the separation factor (*R_L_*) was calculated using Equation (9). In this study, *R_L_* values were found to range between 0.018 and 0.289, indicating highly favorable adsorption. Notably, values of *R_L_* close to zero suggest a strong affinity between the adsorbent and the adsorbate, resulting in a process that is not only efficient but also potentially difficult to reverse under standard conditions.

The *q_m_* value obtained in this study is of the same order of magnitude as those reported by other authors for the adsorption of MB onto palm leaf biomass waste (0.91 mg g^−1^ [47]), alginate beads impregnated with magnetic Chitosan@Zeolite nanocomposite (6.14 mg g^−1^ [48]), avocado seed activated carbon (9.60 mg g^−1^ [9]), or activated carbon from almond shell, walnut shell, hazelnut shell, and apricot stone (1.33 mg g^−1^, 3.53 mg g^−1^, 4.11 mg g^−1^ and 8.82 mg g^−1^, respectively [49]). However, other studies report significantly higher *q_m_* values; for example, when using acid-treated kenaf fiber char (18.18 mg g^−1^ [50]), or rice husk biochar (32.81–33.28 mg g^−1^ [51]). Although the adsorption capacity of biochar derived from exhausted olive pomace (EOPB) may not rank among the highest reported values, its outstanding methylene blue removal efficiency (approaching 100%), together with its low production cost, widespread availability, and minimal environmental impact, make it a viable and sustainable alternative for practical dye removal applications. In addition, the growing implementation of biomass pyrolysis and gasification systems across Europe and the Mediterranean region, driven by circular economy strategies and energy transition policies, is expected to generate substantial quantities of EOPB as a by-product [52].This development may ensure a stable and low-cost supply of biochar with high removal efficiency for both organic and inorganic pollutants in aqueous media. Therefore, the convergence of biomass thermochemical conversion technologies and increasing environmental remediation demands positions EOPB as a highly promising and sustainable adsorbent for future large-scale applications in wastewater treatment.

**Figure 8:**
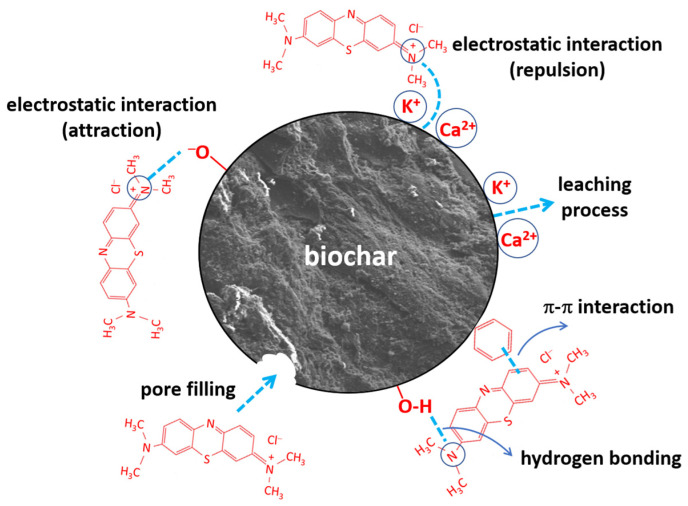
Possible mechanisms for methylene blue adsorption onto EOP biochar.

Regarding the adsorption mechanism of methylene blue (MB) onto biochar, it is a particularly complex process, as multiple interactions typically occur between the adsorbent and the adsorbate, such as hydrogen bonding, π–π interactions, electrostatic attractions, and pore-filling adsorption [53]. In general, both the exhausted olive pomace and its derived biochars exhibited low adsorption capacities, which can be attributed to the high surface content of K^+^ and Ca^2+^ cations that hinder the retention of cationic dyes due to electrostatic repulsion forces. Nevertheless, the slow release of these cations into the liquid phase, by leaching, throughout the adsorption process could partially contribute to MB retention, which may also account for the low adsorption kinetics observed during the experiments. In this regard, the biochars obtained at 300 °C, 400 °C, and 500 °C (with a residence time of 1 h) demonstrated notable cation exchange capacities, as evidenced by the pH increase observed during the adsorption experiments (Figure 1a). During the adsorption process, oxygen-containing functional groups played a key role by enabling attractive interactions, both electrostatic and hydrogen bonding. Additionally, aromatic functional groups contributed to π–π interactions between the adsorbent and the adsorbate. Furthermore, pyrolysis led to a slight increase in the specific surface area of the solid, which could also have a positive effect on dye adsorption. The fact that the density of aromatic functional groups increases with pyrolysis temperature, while the density of oxygen-containing functional groups decreases, resulted in maximum adsorption being achieved with the biochar produced at 400 °C for 1 h. The presence of different types of adsorbent–adsorbate interactions was evidenced by the observation (Section 2.3) that higher agitation speeds led to a decrease in the percentage of MB adsorbed, which could be attributed to the destabilization of π–π interactions, the weakest among the interaction types involved. Based on the above, the proposed MB adsorption mechanism is summarized in Figure 8.

### 2.6. Desorption Study

Desorption is the reverse process of adsorption, involving the removal of adsorbed molecules from the surface of the solid using various desorbing agents. From an economic perspective, recycling the adsorbent plays a key role by helping to reduce material costs. Therefore, it is essential to examine the desorption process of methylene blue adsorbed on EOPB. In alignment with sustainability objectives, the desorption study was limited to water, ethanol, and acetic acid as eluents to mitigate the generation of hazardous leachates [10]. In parallel, the solubility of methylene blue in ethanol and water makes their use effective for dye desorption from adsorbents [54]. Adsorption efficiency was observed to be significantly higher under alkaline conditions; these findings suggest that introducing an acidic solution during the regeneration phase can effectively desorb the adsorbed species.

**Figure 9:**
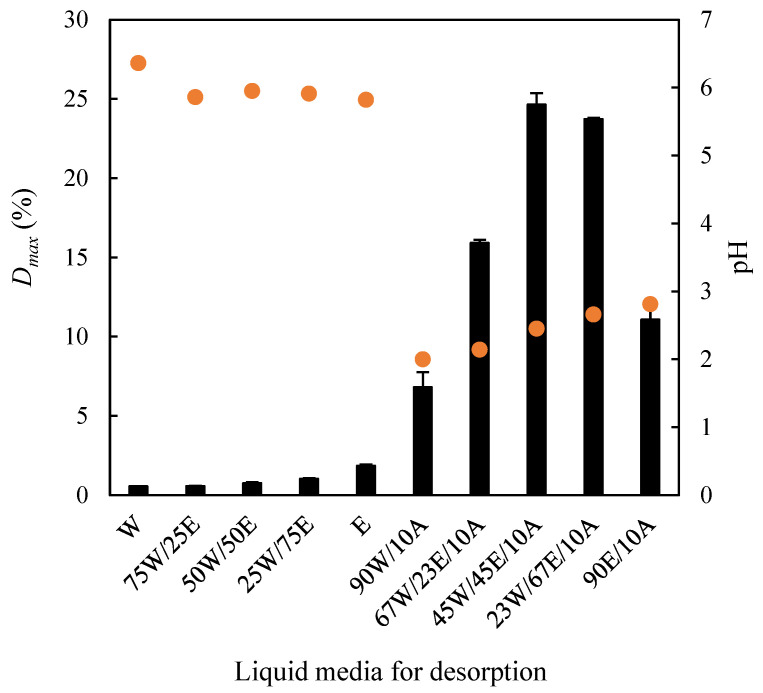
Influence of different liquid media on the desorption of methylene blue adsorbed on the EOPB surface. Bars: desorption percentage. Dots: pH of the desorbing medium. W: water; E: ethanol; A: acetic acid.

Accordingly, the desorption capacity of water, ethanol, and their mixtures was first examined in terms of the maximum desorption percentage (*D_max_*) of methylene blue. Subsequently, the effect of adding 10% (*v*/*v*) acetic acid to the desorbing medium on *D_max_* was assessed. These experiments were conducted at 75 rpm and 30 °C. As illustrated in Figure 9, the desorption of methylene blue was negligible when using water, ethanol, or their binary mixtures, with *D_max_* values ranging from 0.56% (using water, pH 6.36) to 1.85% (using ethanol, pH 5.82). The low desorption capacity of water is consistent with the low *R_L_* values derived from the Langmuir isotherm discussed in Section 2.4. Conversely, the incorporation of 10% acetic acid into the liquid phase led to a marked pH reduction, which significantly enhanced desorption efficiency, yielding a maximum *D_max_* of 24.6% for the ternary mixture composed of 45% water, 45% ethanol, and 10% acetic acid (pH 2.45).

**Figure 10:**
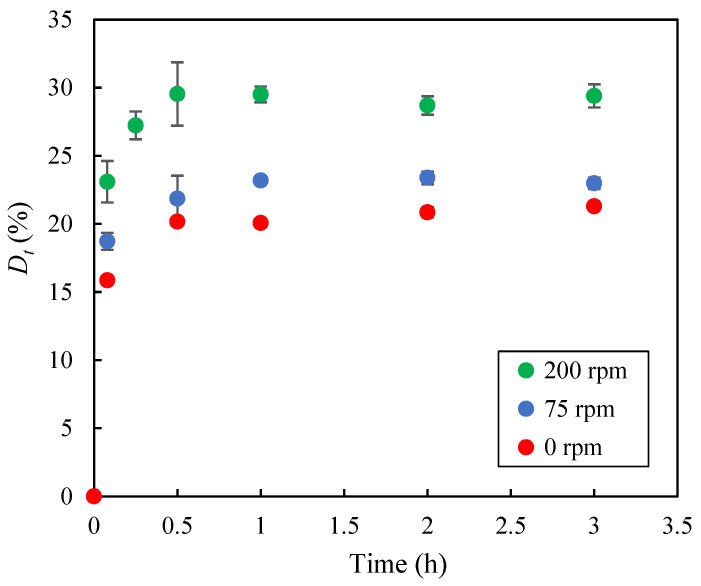
Time evolution of the desorbed methylene blue percentage at 30 °C using the 45W/45E/10A desorbing medium under different agitation speeds.

In addition, the influence of agitation speed on methylene blue desorption was investigated using the 45W/45E/10A medium at 30 °C under three agitation conditions: 0 rpm, 75 rpm, and 200 rpm. As shown in Figure 10, the desorption values (*D_t_*, %), calculated according to Equation (3), increased sharply during the first 0.5 h of the experiments and then stabilized. Furthermore, the highest agitation speed (200 rpm) resulted in the highest desorption values, with a maximum desorption of approximately 30%. To further enhance dye recovery from the biochar, the washing procedure with the 45W/45E/10A medium was repeated four consecutive times under the same conditions (30 °C, 200 rpm). This led to a cumulative *D_max_* of 51.6%. These results suggest that incomplete desorption may be due to strong electrostatic interactions and hydrogen bonds formed between the adsorbate molecules and the functional groups on the EOPB surface and, therefore, further research is required to enhance desorption efficiency and improve the recovery of the solute retained on the biochar.

## 3. Materials and Methods

### 3.1. Raw Material and Chemicals

The pelletized exhausted olive pomace (EOP) used in the present study was kindly provided by the company Daniel Espuny, S.A.U., located in Estación Linares-Baeza, Jaén, Spain (UTM coordinates: 38°03′57.68″ N, 3°35′17.65″ W). The pellets had an average diameter of 5.9 ± 0.1 mm and an average length of 24.2 ± 1.5 mm. The biomass was air-dried in the laboratory until it reached an equilibrium moisture content of 7.19 ± 0.11%. Afterwards, it was stored in plastic bags until further characterization and use in pyrolysis experiments. The chemical reagents employed were methylene blue dye (MB, purity 82%), ethanol (99.8%, *v*/*v*), and acetic acid (99.7%, *w*/*w*), all supplied by Panreac, while the water used was deionized water.

### 3.2. Pyrolysis Treatment

**Figure 11:**
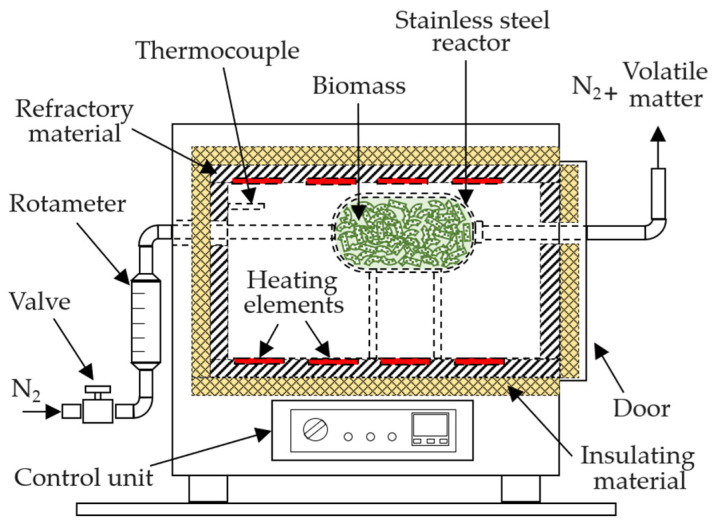
Equipment used for biomass pyrolysis.

The pelletized exhausted olive pomace was subjected to thermal treatment in a stainless-steel reactor with a total capacity of 1 L, placed inside a muffle furnace (Selecta, Mod. Select-Horn-TFT, with a total internal volume of 9 L, Figure 11). The reactor was loaded with approximately 320 g of biomass. To ensure non-oxidizing conditions during the experiments, a continuous nitrogen flow of 400 mL min^−1^ was introduced into the reactor, starting 10 min before heating and maintained until the end of the thermal process. A heating rate of 10 °C min^−1^ was applied in all experiments. Once the target temperature was reached, it was held constant for a defined holding time. After this period, the reactor was sealed, removed from the furnace, and rapidly cooled with water, reducing its temperature to below 100 °C in less than 5 min. The thermally treated biomass (biochar) was then left at room temperature for at least four days to reach equilibrium moisture content before being stored in plastic bags.

### 3.3. Adsorption and Desorption Experiments

Adsorption experiments were carried out in 0.1 L Erlenmeyer flasks with airtight plastic seals, placed inside an IKA INC 125 FS Digital Incubator Shaker, which allowed for the control of temperature and agitation speed. In this study, the pH of the liquid medium was not controlled in order to reduce reagents consumption and enhance the potential economic feasibility of the adsorption process. It is known that methylene blue adsorption generally does not significantly improve when working at pH values above 7 [55,56]. In this regard, in the experiments conducted in this study, where methylene blue concentrations ranged from 5 to 200 mg L^−1^, the initial pH of those solutions varied between 7.1 and 6.8, respectively. Samples were withdrawn at different times throughout the adsorption process from the Erlenmeyer flasks to spectrophotometrically determine the time evolution of the solute concentration in the aqueous medium. In this way, the percentage of methylene blue adsorbed at a given time *t* (*A_t_*, %) could be calculated using Equation (1).

(1) $$A_{t}\;(\%)=\frac{C_{0}{-\;C}_{t}}{C_{0}}\times 100$$

where *C*_0_ and *C_t_* are the concentrations of methylene blue (mg L^−1^) at the initial time and at a given time (*t*), respectively. When the methylene blue concentrations in the liquid phase remained constant, it was considered that equilibrium had been reached, allowing the maximum adsorption percentages (*A_max_*, %) to be determined in the experiments. On the other hand, the adsorption capacity at time *t* (*q_t_*, mg g^−1^) was determined using Equation (2),

(2) $$q_{t}=\frac{0.05{\;(C}_{0}{-\;C}_{t})}{m}$$

where 0.05 is the volume (L) of methylene blue solution in the Erlenmeyer flask, *C*_0_ and *C_t_* are the initial and time-dependent solute concentrations (mg L^−1^), respectively, and *m* (g) is the mass of the adsorbent solid used (on a dry basis). Equation (2), when applied at the equilibrium time, allows the calculation of the maximum adsorption capacity (*q_max_*, mg g^−1^) for a given experiment.

Once the adsorption of methylene blue onto the bioadsorbent was completed, desorption experiments were carried out by recovering the solids, via vacuum filtration, and subsequently placing them in 0.1 L Erlenmeyer flasks together with 0.05 L of various liquid media (water, W; ethanol, E; water–ethanol mixtures, W/E; water–acetic acid mixtures, W/A; ethanol–acetic acid mixtures, E/A; and water–ethanol–acetic acid mixtures, W/E/A). Desorption was performed in an orbital shaker (IKA INC 125 FS digital). At different time intervals, liquid-phase samples were collected to determine the amount of methylene blue transferred from the solid. In this way, the desorption percentage at a given time *t* (*D_t_*, %) could be determined using Equation (3).

(3) $$D_{t}\;(\%)=\frac{0.05{\;C}_{D}}{m_{0}}$$

where 0.05 is the volume (L) of the liquid medium, *C_D_* is the concentration (mg L^−1^) of methylene blue in the liquid phase at time *t*, and *m*_0_ (mg) is the mass of methylene blue initially adsorbed onto the bioadsorbent at the start of the desorption experiment. When methylene blue concentrations in the liquid medium remained constant, equilibrium was assumed to have been reached, and the maximum desorption percentages (*D_max_*, %) could then be determined. To enhance desorption, in some experiments multiple sequential desorption stages were performed by contacting the bioadsorbent with fresh liquid medium following a cross-current contact approach. For this purpose, in each stage, the solid recovered by vacuum filtration from the previous desorption step was placed again in a 0.1 L Erlenmeyer flask together with 0.05 L of fresh liquid medium.

All adsorption and desorption experiments were carried out at least in duplicate, with standard deviations always below 9% of the mean values.

### 3.4. Kinetic Study and Adsorption Isotherms

The time evolution of the amount of methylene blue retained on the surface of the bioadsorbents was fitted to four commonly used adsorption kinetic models, pseudo-first order, pseudo-second order, Weber–Morris, and Elovich [57,58], whose linearized forms correspond to Equations (4), (5), (6), and (7), respectively.

*ln* (*q_e_* − *q_t_*) = *ln q_e_* − *K*_1_ *t* (4)

(5) $$\frac{t}{q_{t}}=\frac{1}{K_{2}q_{e}^{2}}+\frac{1}{q_{e}}\;t$$

(6) $${\ln q}_{t}{=\ln K}_{\mathrm{df}}+\frac{1}{2}\ln t$$

(7) $$q_{t}=\frac{ln\left(\alpha \beta \right)}{\beta }+\frac{ln\;t}{\beta }\;$$

where *q_e_* (mg g^−1^) is the equilibrium adsorption capacity, and *K*_1_ (g mg^−1^ h^−1^), *K*_2_ (g mg^−1^ h^−1^), *K_df_* (mg g^−1^ h^−0.5^), *α* (mg g^−1^ h^−1^), and *β* (g mg^−1^) are the equilibrium constant rates corresponding to Equations (4), (5), and (6), respectively.

On the other hand, the experimental equilibrium data (*q_e_* and *C_e_*) were fitted to the four adsorption isotherms shown below:

- Langmuir isotherm, Equation (8):

(8) $$q_{e}=\frac{q_{m}{\;K}_{L\;}{\;C}_{e}}{1+\;K_{L}{\;C}_{e}}$$

where *q_m_* (mg g^−1^) is the maximum adsorption capacity, *C_e_* (mg L^−1^) is the equilibrium methylene blue concentration, and *K_L_* (L mg^−1^) is the adsorbent–adsorbate equilibrium constant. Based on the *K_L_* value, the separation factor, or equilibrium parameter (*R_L_*), a dimensionless constant, can be calculated using Equation (9):

(9) $$R_{L}=\;\frac{1}{1+\;K_{L}C_{0}}$$

where *C*_0_ (mg L^−1^) is the initial solute concentration.

- Freundlich isotherm, Equation (10):

(10) $$q_{e}=K_{F}{\;C}_{e}^{1/n}$$

where *n* is a dimensionless adsorption coefficient, which characterizes the intensity, while *K_F_* (mg g^−1^) (mg L^−1^)^−1^/*n* is the Freundlich constant related to the adsorption capacity and *C_e_* (mg L^−1^) is the methylene blue concentration of the solution at equilibrium.

- Temkin isotherm, Equation (11):

*q_e_* = *B ln*(*K_T_ C_e_*) (11)

where *B* = *q_m_*R*T*/*b_T_*, *q_m_* (mg g^−1^) is the maximum adsorption capacity, R is the gas constant (8.3145 J mol^−1^ K^−1^), *T* is the absolute temperature (K), *b_T_* (J mol^−1^) is a constant related to the heat of the adsorption, *K_T_* (L mg^−1^) is the equilibrium-binding constant, and *C_e_* (mg L^−1^) is the methylene blue concentration of the solution at equilibrium.

- Dubinin–Radushkevich isotherm, D-R, Equation (12):

(12) $$q_{e}=q_{m}{\;e}^{{-K}_{\mathrm{DR}}{\;\varepsilon }^{2}}$$

where *K_DR_* (mol^2^ J^−2^) is the Dubinin–Radushkevich constant, related to mean free energy of adsorption, and *ε* is the Polanyi potential (J mol^−1^) which is equal to [R *T ln* (1 + 1/*C_e_*)].

To assess the validity of the proposed models (kinetic and equilibrium), both the coefficient of determination (*R*^2^) and the sum of error values (*SE*, %) were calculated according to Equations (13) and (14).

(13) $$R^{2}=1-\;\frac{\sum {\left(q_{\exp}-\;q_{\mathrm{cal}}\right)}^{2}}{\sum {\left(q_{\exp}-\;{q_{\mathrm{cal}}}^{\mathrm{ave}}\right)}^{2}}$$

(14) $$SE\;\left(\%\right)=\frac{100}{N-1}\;\sum \frac{abs\left(q_{exp}-q_{cal}\right)}{q_{exp}}\;$$

where *q_exp_* and *q_cal_* are the experimental and predicted adsorption capacities data points, respectively, *q_cal_^ave^* is the average value of the experimental data and *N* is the number of data points.

### 3.5. Analytical Methods

The concentration of MB was determined using a UV–vis spectrometer (BIOCHROM mod. Libra S60; wavelength of 664 nm) that was precalibrated with MB solutions within in the range 0.1–10 mg L^−1^. Proximate and ultimate analyses of the biomasses were performed according to Karim et al. [59]. The surface morphology of the solid samples was examined using a high-resolution scanning electronic microscope (FE-SEM, Zeiss SUPRA40VP, Zeiss Co., Oberkochen, Germany). The equipment allowed elemental microanalysis by Energy Dispersive X-ray Spectroscopy (EDX) using a large-area X-Max 50 mm detector. Additionally, nitrogen porosimetry was performed to determine both the porosity and specific surface area (Micromeritics ASAP 2020, Norcross, GA, USA). FTIR spectra were recorded in the 4000–400 cm^−1^ range using a Vertex 70 spectrometer (Bruker, Billerica, MA, USA) with the KBr pellet method, a resolution of 4 cm^−1^, and 100 scans. A digital caliper (ABS Digimatic Caliper, 500-182-30, Mitutoyo, Kawasaki, Japan) was used to determine the dimensions of the pelletized exhausted olive pomace and the hydrochar obtained by pyrolysis of the raw material.

## 4. Conclusions

Exhausted olive pomace (EOP) presents a high mineral matter content, with K^+^ and Ca^2+^ cations being particularly abundant. These cations negatively affect the adsorption of methylene blue (a cationic dye) through electrostatic repulsion. The transformation of EOP into biochar at low temperatures (200–500 °C) induces changes in the specific surface area and functional groups of the biomass, resulting in maximum adsorption when using the biochar produced at 400 °C for 1 h. This is attributed to a suitable balance in the sample between mineral matter and oxygen-containing and aromatic functional groups. Optimization of adsorption parameters such as particle size (2–3 mm), agitation speed (75 rpm), and solid loading (1 g per 0.05 L of MB solution) allowed for dye removal efficiencies close to 100%. The kinetic study revealed that the data fitted a pseudo-second-order model, with low initial adsorption rates, which could be explained by the fact that the retention rate of the dye is mainly governed by the transfer of cations from the solid to the liquid phase through a leaching process. The equilibrium study at 30 °C indicated that MB adsorption onto EOPB follows a Langmuir isotherm, reflecting strong adsorbent–adsorbate interactions, as evidenced by low *R_L_* values. Consequently, dye desorption is very limited when using water as the extracting solvent; however, methylene blue recovery can be significantly improved (up to 52%) by combining an agitation speed of 200 rpm with a liquid medium consisting of 45% water, 45% ethanol, and 10% acetic acid. In any case, further studies are needed to enhance the recovery of the solute retained on the biochar.

## Figures and Tables

**Table 1:** Proximate and ultimate analyses (dry basis) of exhausted olive pomace (EOP) and its derived biochars obtained at 400 °C and 500 °C (1 h holding time).

Parameter		EOP	Biochar 400 °C	Biochar 500 °C
Proximate analysis	Ash (%)	9.5 ± 0.3	21.5 ± 0.3	25.6 ± 0.1
	Fixed carbon * (%)	15.7 ± 1.2	46.7 ± 1.6	44.0 ± 1.4
	Volatile matter (%)	74.8 ± 1.2	31.9 ± 1.3	30.4 ± 1.5
Ultimate analysis	C (%)	43.5 ± 0.2	56.1 ± 1.2	61.1 ± 0.8
	H (%)	5.6 ± 0.0	3.9 ± 0.0	2.6 ± 0.1
	N (%)	1.6 ± 0.0	1.8 ± 0.0	1.3 ± 0.0
	S (%)	1.1 ± 0.5	0.5 ± 0.3	0.6 ± 0.1
	O * (%)	48.2 ± 0.2	37.8 ± 1.6	34.3 ± 0.7

* By difference.

**Table 2:** Kinetic parameters for methylene blue (MB) adsorption onto biochar produced at 400 °C for 1 h according to pseudo-first order (PFO), pseudo-second order (PSO), Weber–Morris (WM), and Elovich kinetic models. Adsorption conditions: biochar load, 1 g per 0.05 L of MB solution; 30 °C; 75 rpm.

Kinetic Models	Initial Solute Concentration (*C*_0_) and Kinetic Parameters						
PFO	*C*_0_ (mg L^−1^)	*q_e,cal_* (mg g^−1^)		*K*_1_ (g mg^−1^ h^−1^)		*R* ^2^	*SE* (%)
	50	1.980		0.133		0.991	36.0
	100	3.973		0.104		0.992	35.7
PSO	*C*_0_ (mg L^−1^)	*q_e,cal_* (mg g^−1^)		*K*_2_ (g mg^−1^ h^−1^)		*R* ^2^	*SE* (%)
	50	2.667		0.107		1.000	2.3
	100	5.298		0.039		1.000	3.4
WM	*C*_0_ (mg L^−1^)	*Kdf*_1_^(1)^	*R* ^2^	*SE* (%)	*Kdf*_2_ ^(1)^	*R* ^2^	*SE* (%)
	50	0.721	0.993	5.3	0.268	0.991	9.9
	100	1.165	0.994	4.9	0.451	0.985	1.7
Elovich	*C*_0_ (mg L^−1^)	*α* (mg g^−1^ h^−1^)		*β* (g mg^−1^)		*R* ^2^	*SE* (%)
	50	1.939		1.981		0.965	7.8
	100	2.553		0.950		0.982	6.2

^(1)^ Units: mg g^−1^ h^−0.5.^

**Table 3:** Adsorption isotherms parameters at 30 °C for methylene blue removal onto biochar obtained at 400 °C for 1 h.

Langmuir	*K_L_* (L mg^−1^)	*q_m_* (mg g^−1^)	*R* ^2^	*SE* (%)
	0.269	6.414	0.999	4.25
Freundlich	*K_F_* (mg g^−1^ (mg L^−1^)^−n^)	*n*	*R* ^2^	*SE* (%)
	1.229	2.035	0.890	30.13
Temkin	*K_T_* (L mg^−1^)	*B* (mg g^−1^)	*R* ^2^	*SE* (%)
	4.917	1.113	0.989	9.77
D-R ^(1)^	*q_m_* (mg g^−1^)	*K_DR_* 10^7^ (mol^2^ J^−2^)	*R* ^2^	*SE* (%)
	4.204	1.600	0.905	24.47

^(1)^ Dubinin–Radushkevich isotherm.

## Data Availability

All data and materials described in this work are available in this article. Further inquiries can be directed to the corresponding author.

## Supplementary Materials

The following supporting information can be downloaded at https://www.mdpi.com/article/10.3390/molecules30153254/s1: Figure S1: EDX analysis of EOP; Figure S2: EDX analysis of the biochar obtained at 400 °C × 1 h; Figure S3: EDX analysis of the biochar obtained at 500 °C × 1 h.

## Abbreviations

The following abbreviations are used in this manuscript:

A	Acetic acid
*A_max_*	Maximum adsorption percentage (%)
APD	Average Pore Diameter (Å)
*A_t_*	Adsorption percentage at a given time (%)
*C_e_*	Equilibrium concentration of methylene blue (mg L^−1^)
*D_max_*	Maximum desorption percentage (%)
D-R	Dubinin–Radushkevich isotherm
*D_t_*	Desorption percentage at a given time (%)
E	Ethanol
EOP	Exhausted olive pomace
EOPB	Exhausted olive pomace biochar (obtained at 400 °C for 1 h)
MB	Methylene blue
PFO	Pseudo-first order (kinetic model)
PSO	Pseudo-second order (kinetic model)
*q_e_*	Equilibrium adsorption capacity (mg g^−1^)
*q_max_*	Maximum adsorption capacity (mg g^−1^)
*q_t_*	Adsorption capacity at a given time (mg g^−1^)
*R*^2^	Coefficient of determination
*SE*	Sum of error values (%)
SSA	Specific surface area (m^2^ g^−1^)
W	Water
WG	Without grinding (pelletized biochar with original size)
WM	Weber–Morris (kinetic model)
*α*	Initial sorption rate in the Elovich model (mg g^−1^ h^−1^)
*β*	Constant related to the extent of surface coverage and activation energy for chemisorption in the Elovich model (g mg^−1^)
