# Supplementary material for: Production of Bioadsorbents via Low-Temperature Pyrolysis of Exhausted Olive Pomace for the Removal of Methylene Blue from Aqueous Media
Source: Molecules. 2025 Aug 3;30(15):3254. doi: 10.3390/molecules30153254 (PMC12348985; doi:10.3390/molecules30153254)
Supplement: Supplementary file 1 [file molecules-30-03254-s001.zip › molecules-3760525-supplementary.pdf]

Supplementary Materials

Production of bioadsorbents, via low-temperature pyrolysis of exhausted olive pomace, for the removal of methylene blue from aqueous media

Safae Chafi [1,*], Manuel Cuevas-Aranda [1,2,*], Mª Lourdes Martínez-Cartas [1,2] and Sebastián Sánchez [2]

[1] Department of Chemical, Environmental and Materials Engineering, Science & Technology Campus of Linares, University of Jaén, Avda. de la Universidad s/n, 23700 Linares, Spain.; lcartas@ujaen.es (M.L.M-C)

[2] Olive Grove and Olive Oils Research Institute, ES-23071, Jaén, Spain; ssanchez@ujaen.es (S.S.)

* Correspondence: sc000025@red.ujaen.es (S.C.); mcuevas@ujaen.es (M.C-A.)

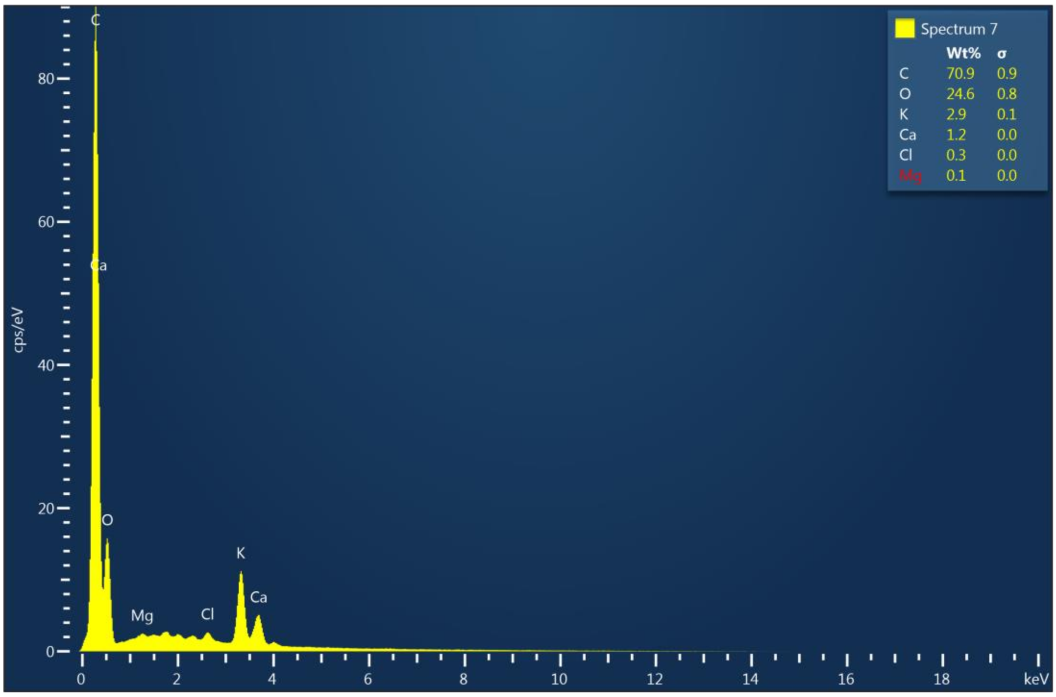

Figure S1. EDX analysis of EOP.

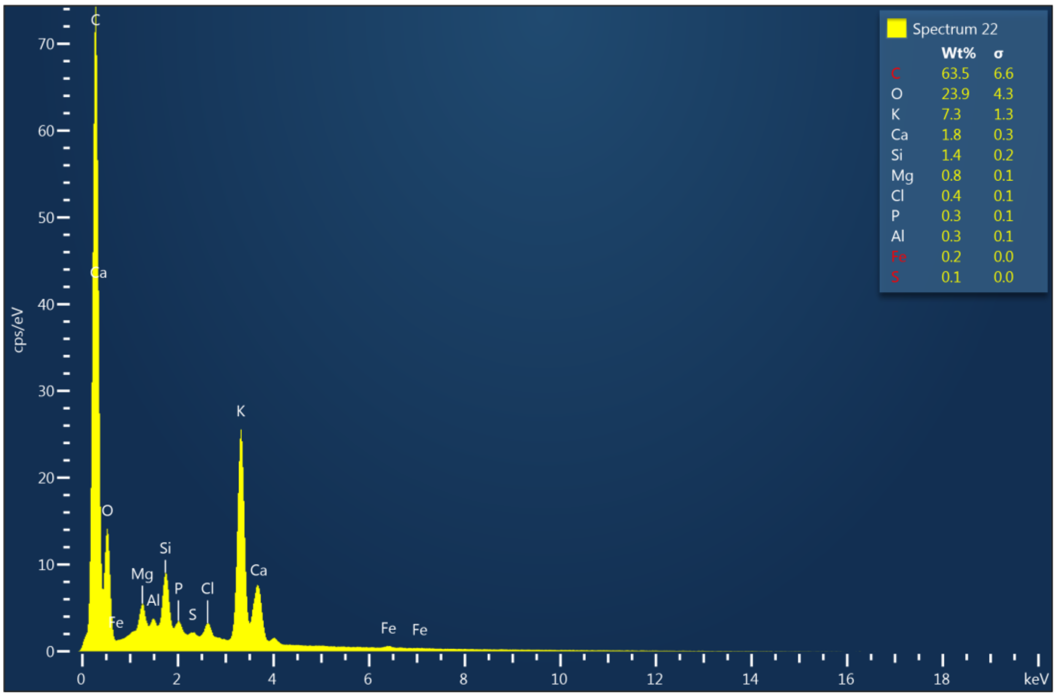

Figure S2. EDX analysis of the biochar obtained at 400 °C x 1 h.

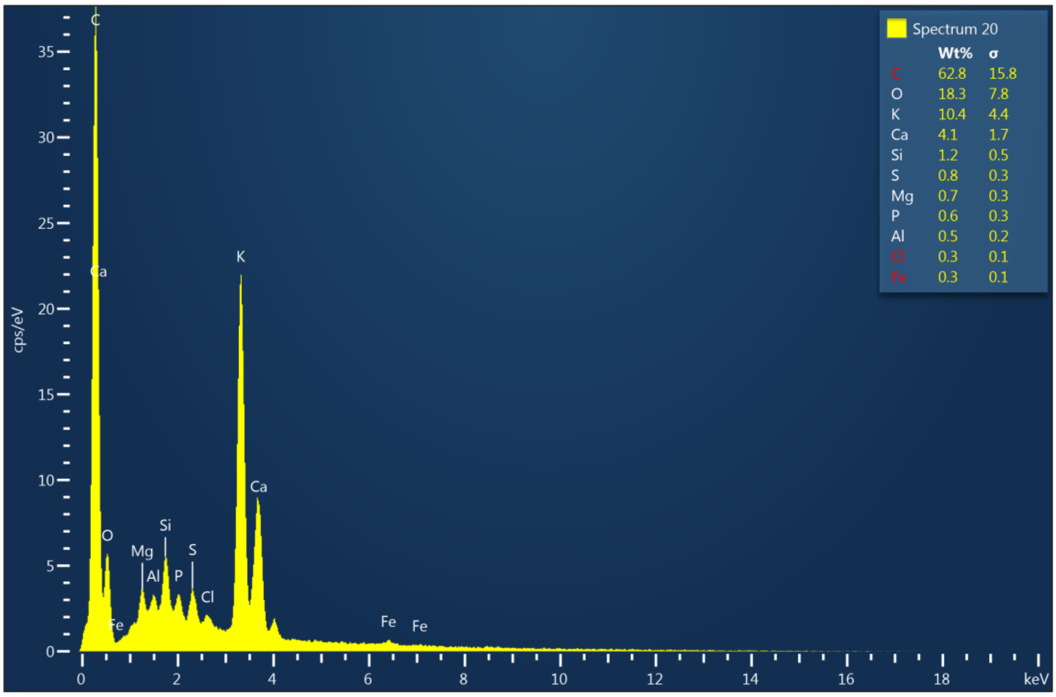

Figure S3. EDX analysis of the biochar obtained at 500 °C x 1 h.
